# Lateralized Readiness Potentials Reveal Properties of a Neural Mechanism for Implementing a Decision Threshold

**DOI:** 10.1371/journal.pone.0090943

**Published:** 2014-03-13

**Authors:** Marieke K. van Vugt, Patrick Simen, Leigh Nystrom, Philip Holmes, Jonathan D. Cohen

**Affiliations:** 1 Department of Artificial Intelligence, University of Groningen, Groningen, The Netherlands; 2 Department of Neuroscience, Oberlin College, Oberlin, Ohio, United States of America; 3 Princeton Neuroscience Institute, Princeton University, Princeton, New Jersey, United States of America; 4 Department of Mechanical & Aerospace Engineering, Princeton University, Princeton, New Jersey, United States of America; Radboud University Nijmegen, Netherlands

## Abstract

Many perceptual decision making models posit that participants accumulate noisy evidence over time to improve the accuracy of their decisions, and that in free response tasks, participants respond when the accumulated evidence reaches a decision threshold. Research on the neural correlates of these models' components focuses primarily on evidence accumulation. Far less attention has been paid to the neural correlates of decision thresholds, reflecting the final commitment to a decision. Inspired by a model of bistable neural activity that implements a decision threshold, we reinterpret human lateralized readiness potentials (LRPs) as reflecting the crossing of a decision threshold. Interestingly, this threshold crossing preserves signatures of a drift-diffusion process of evidence accumulation that feeds in to the threshold mechanism. We show that, as our model predicts, LRP amplitudes and growth rates recorded while participants performed a motion discrimination task correlate with individual differences in behaviorally-estimated prior beliefs, decision thresholds and evidence accumulation rates. As such LRPs provide a useful measure to test dynamical models of both evidence accumulation and decision commitment processes non-invasively.

## Introduction

Decision making has traditionally been described as a process of evidence accumulation up to an abstract decision threshold, in which accumulation over time increases the chance of an accurate response (e.g., [1]). Here we focus on a mechanism for implementing this decision threshold in a neurally-plausible network, and the predictions that makes for neural activity [2], [3]. We then show that these predictions are satisfied by the lateralized readiness potential (LRP), a difference wave between centrally located scalp potentials that routinely accompanies manual responding. Paradoxically, this emphasis on decision threshold activity turns out to provide a useful methodological approach to studying the evidence accumulation process thought to precede decision threshold-crossing.

A canonical evidence accumulation model for response-time tasks is the drift diffusion model (DDM; [4]). In the DDM, the presentation of a stimulus drives a noisy evidence-accumulation process until it reaches one of two decision thresholds. Participants emit the response corresponding to the threshold reached. The quality of perceptual information determines the speed of accumulation (drift) relative to the intensity of background noise (diffusion). The noisiness of the process accounts for across-trial variability in response times (RTs) and accuracy. Differences in the height of the decision threshold determine speed-accuracy trade-offs (SATs): an increase in threshold height emphasizes accuracy over speed. A participant's accuracy is determined by which of the decision thresholds is reached first. His/her response time (RT) is determined by the time it takes to reach this decision threshold, plus the time taken for non-decision processes (e.g., perceptual and motor delays).

Recent efforts have focused on finding neural correlates of the process of evidence accumulation in perceptual decision making with monkey neurophysiology (e.g., [5]–[7]), human magnetoencephalography (e.g., [8], [9]), functional magnetic resonance imaging (fMRI; e.g., [10]) and electroencephalography (EEG; e.g., [11]–[13]). Although neural correlates of decision thresholds and changes in their height have been extensively studied [14]–[17], decision making research typically treats the crossing of a decision threshold abstractly. In other words, it is unclear how the system moves from decision-preparation/evidence accumulation (OFF) instantaneously to decision-commitment (ON) [18]. In the preparation state, the system sends virtually zero input to the motor system until evidence builds to a critical level, at which point a punctate transition into the decision-commitment state occurs, possibly accompanied by an almost immediate muscular contraction [19]. Consequently most decision making models implicitly assume that the decision system is capable of implementing a non-linear step function that switches from OFF to ON when evidence exceeds some critical level. Subcortical structures are good candidates for such punctate transitions [20], [21] but what if the transition is not in fact punctate, but is instead a relatively gradual process in its own right? Would cortical populations with the same properties (i.e., time constants) as cortical accumulators be able to implement such a threshold mechanism, or must such mechanisms be relegated to subcortical structures such as the basal ganglia?

To address this question, we used a multi-layer neural network model that explicitly describes how accumulated evidence is transformed into a motor response [2], [3]. We previously hypothesized that the threshold layer of this model was associated with premotor cortex. Since this is the primary source of the LRP that can be measured with scalp EEG, our objective was to examine whether changes in LRP shape and amplitude across task conditions support and confirm the qualitative predictions of our model. If so, this would lend support to a model of cortical decision commitment.

### Specification of the multi-layer decision model

The circuit model that describes the complete process of evidence accumulation and motor implementation [2], [3] is based on a simplified representation of firing-rate activity in a cortical neural population (cf. [22], [23]). We do not explicitly model how this population-level activity translates into macroscopically observable EEG activity via forward modeling with a skull and scalp model since we do not have enough physical information to justify this approach. We rather propose a qualitative mapping in which increases and decreases in model activity are reflected in similar changes in the across-trial average of event-locked EEG signals, the event-related potential (ERP). Under this representation, neural populations typically act as leaky integrators of the activity of their input populations.

A more detailed model description, including equations, can be found in File S1. Simply, the model makes use of the fact that recurrent units can be configured such that they have exactly two stable states, reflecting the two states of decision preparation and commitment. These are exactly the same units that can be configured to work as integrators when the recurrent connections are weak enough.

### Activation profiles of threshold detectors

**Figure 1:**
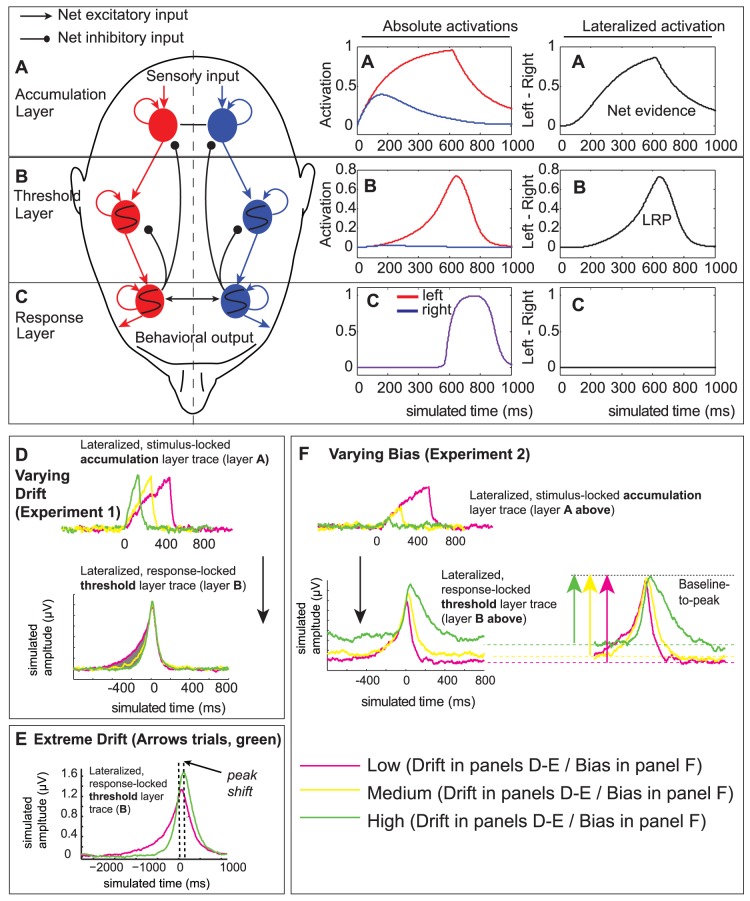
Neural network implementation of the DDM and associated LRP predictions. The neural network model of decision making consists of three layers (units with S-shaped curves denote bistable switch units). In the evidence accumulation layer (A), located potentially in parietal cortex, activity increases with motion input over time (red = leftward motion; blue = rightward motion). The difference in left minus right activations approximates a drift-diffusion process. This accumulation layer feeds into the threshold layer (B), which is potentially located in primary motor cortex. The difference between left and right switch unit activations then reflects the LRP signal. The threshold layer's output is transformed into a punctate motor output in the response layer (A), which also ensures that the threshold and accumulation layers are reset after the response. (D) Our model predicts that for changes in signal-to-noise ratio, in the accumulation layer the slope of neural activity changes, while in the threshold layer there is a non-linear change in slope. Consequently, the change due to increases in signal-to-noise ratio is best quantified by the area between curves. (E) Additionally, the model predicts that the magnitude and location of the response-locked simulated LRP should vary with signal-to-noise ratio (pink = low drift;green = high drift). (F) When varying response bias, the model predicts that the height of the LRP should change (pink = low bias;green = high bias).

Putting together these bistable units with integrator units, Simen and Cohen (2009) [2] created a model for two-alternative forced choices. This model consisted first of an evidence accumulation layer consisting of two leaky competing accumulators (cf. [24]), each of which accumulates evidence in favor of one of two competing response options. Mounting evidence suggests that evidence accumulation is mediated by a large variety of brain areas, such as parietal cortex (e.g., [5]), dorsolateral prefrontal cortex [25], [26], frontal eye field–FEF [27], [28], caudate [29], and superior colliculus [30]. We therefore depict evidence accumulation in somewhat posterior, bilateral electrode locations, where evidence for, say, a left button press is integrated in the accumulation layer in the right hemisphere (blue) and evidence for a right button press is similarly integrated in the left hemisphere (red). The middle panel of Figure 1A shows the activation of each accumulator unit over time; the right panel of Figure 1A plots the difference between these activations. This difference approximates a drift-diffusion process when the leakiness of the accumulators is balanced by reciprocal, lateral inhibition between them [31].

The second layer (Figure 1B; blue shading) is the threshold layer that consists of the bistable switch units described above, which detect when activity in either of the corresponding accumulation layer units has exceeded a critical value. The threshold units are leaky integrators with strongly positive, recurrent feedback. This positive feedback causes the units to produce nearly binary outputs which can remain relatively quiescent for a broad range of inputs up to one critical value, at which point they quickly become maximally activated and remain so even for modest fluctuations in their input (see, e.g., [22], [23]).

The middle panel of figure 1B shows threshold-unit activations and the right panel of Figure 1B shows their difference over time. Threshold units track the accumulation units in the period prior to the stereotyped peak. The pre-stimulus level and the rate of increase of threshold-unit activity after stimulus onset can be modulated by biasing signals sent to the threshold units. These signals can adapt SAT and response-bias settings to take advantage of changes in task conditions that affect reward rate [32].

Finally, like the threshold layer, the response layer (Figure 1C) consists of interconnected, bistable switch units that implement a ‘reset’ signal. They activate at the time of a response and, in addition to triggering movement, provide feedback inhibition that drives down the accumulator and threshold unit activations in preparation for the next decision.

While there have been many studies of the neural correlates of evidence accumulation and motor responses, very little is known about the neural correlates of the process that connects these two: threshold crossing (but see [13]). We propose that the dynamics of the threshold layer may be reflected in LRP activity. This is in contrast to previous work, which has suggested that either the LRP reflects something akin to our evidence accumulation layer [33], or something more akin to our response layer [34], [35]. Because neither of these views has been conclusive, we propose a perspective that could reconcile them. Since the threshold layer lies between the response and accumulation layers, it has both a continuous and a ballistic component to its dynamics. The gradual aspect is in agreement with Spencer & Coles' conception [33], while the ballistic aspect is in agreement with Rinkenauer et al.'s conception [35]. Note that we expect only a qualitative match between model-activations and LRPs, since each unit in our model represents a population of neurons, and the model does not contain detailed assumptions about brain anatomy and filtering of neural activity by the skull.

In particular, we predict that the slow, early part of the LRP should display correlations with the participants' rate of evidence accumulation, which can be conveniently estimated with a DDM fit. This correlation with drift rate arises because changes in threshold-unit activation depend strongly on the threshold unit's input (see Figure 1D). In other words, as drift varies, the difference between preliminary activations across conditions forms the gray area depicted in Figure 1D. Specifically, changes in drift should produce a positive area between the LRP curves when the high-drift LRP is subtracted from the low-drift LRP, and the size of this area should be correlated with the difference in drift values estimated from fits of the DDM to RT and accuracy data. We focus on the area between curves rather than rate of rise of the function because the area between curves is much less sensitive to artifactual fluctuations in the EEG data [36].

This account makes a further prediction. Very high levels of drift can cause an overshoot phenomenon, in which the threshold unit activation rises to a higher maximum before resetting, relative to lower-drift conditions (see Figure 1E). As a result, the peak of the LRP moves in position, and shifts closer to the time of the response for very high drift rates. This peak-location prediction is interesting because although it has been observed in the LRP literature [35], we are unaware of any widely accepted account of it.

In contrast to the slow early part of the LRP, the fast, stereotyped part of the LRP should depend mostly on the threshold unit's self-excitation properties. This means it should exhibit a rapid ballistic increase once a critical value of input has been surpassed. Changes in the magnitude of this ballistic component (relative to baseline levels of activation) result primarily from strategic biasing. This biasing changes response probabilities prior to evidence accumulation by adding a continuous, biasing increment to the accumulated evidence that otherwise constitutes the threshold unit's input; this biasing in turn elevates initial activation levels. Figure 1F shows three different levels of response bias: for the pink plot, almost no additional bias is added; for the yellow, an intermediate level of input bias is added, with the result that the pre-stimulus input level hovers to the right of zero. The corresponding output level increases relative to the zero-bias case but remains at the low-activation stable state. The green trace shows the effect of a strong bias, which brings the activity of the threshold unit close to its tipping point.

Empirically, changes in response bias should therefore affect the baseline-to-peak height of the LRP. Hence, differences in LRP-peak amplitude across bias conditions should be correlated with differences in the estimated starting point parameter of the DDM. We will test the predictions for signal-to-noise ratio and response bias in two experiments.

## Results

### Experiment 1

**Figure 2:**
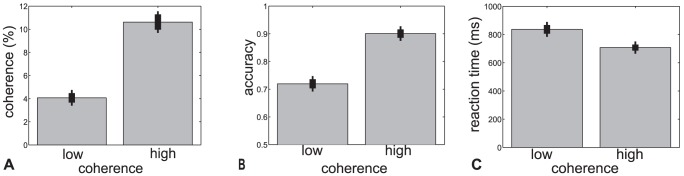
Behavioral performance in Experiment 1. Mean (sem) coherence (A), accuracy (B), and response time (C) across subjects for the low and high coherence conditions. Coherences were tuned to ensure approximately 70 and 90% correct performance for each participant, and the two conditions are statistically different from each other for all three measures (coherence, accuracy, and response time).

Our first prediction concerns the effect of motion coherence on the LRP. Stimulus coherence affects the speed of evidence accumulation in the model (see Figure 1D). This accumulation rate is reflected in threshold layer activations primarily by the rise time of the predicted LRP, resulting in a separation between the curves for different coherence levels. As the difference between stimulus coherences increases, the separation between these curves should increase. Before testing this prediction experimentally, we wanted to confirm that our manipulation of coherence was successful. Figure 2 illustrates the coherences used for the two difficulty levels (A) and the resulting behavioral data (B,C). As expected, accuracy was lower [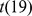 = 18.0, 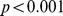] and RT was longer [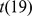 = 9.0, 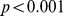] for the more difficult low-coherence trials, which had a significantly lower coherence than the high-coherence trials [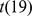 = 15.0, 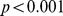].

We also used two control tasks to test whether putative threshold-crossing activity reflected in the LRP from the dot-motion task reflected evidence accumulation alone, or in contrast, reflected only evidence-independent motor preparation. In these tasks, participants were not required to integrate motion evidence (non-integration tasks). In the *signal detection* control task, participants were instructed to press a pre-defined button (left or right), as soon as any dot-stimuli appeared on the screen. These dot-stimuli had 0% motion coherence. The task was designed to measure participants' signal detection RTs to the appearance of dot-stimuli. These signal detection times were then used to create trials of the second control task, the *arrow task*.

The *arrow task* was designed to be virtually identical to the dot-motion discrimination task, with the exception of the evidence accumulation process. As such, the *arrows* trials and dot-motion trials, which were run in separate blocks, were matched in perceptual and motor demands and average dot-motion viewing time, but the arrows task required only brief, non-noisy evidence accumulation on the part of the participant.

We fit the pure DDM to the behavioral data for each individual participant separately with the Matlab Diffusion Model Analysis Toolbox, DMAT [37], [38]. We allowed the drift, starting point and non-decision time (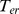) to vary between the low and high coherence conditions, and restricted the variability parameters to be zero. An unbiased starting point is half the magnitude of the decision threshold parameter. Because there is evidence that participants keep their decision thresholds constant when only drift rate is experimentally manipulated [39], we kept thresholds fixed across conditions during fitting. We also tried a model in which the threshold varied between low- and high-coherence conditions, which was a better model (lower Bayesian Information Criterion; BIC) for 8/20 participants. A much more complex model in which drift, threshold, non-decision time were all allowed to vary between conditions and the various noise parameters were also non-zero, was the best model (lowest BIC) for 4/20 participants. We computed separate fits for the arrow and signal detection trials. Following common practice, the noise coefficient in these fits was constrained to be 0.1. Table 1 shows the mean fitted parameters.

**Table 1:** Pure DDM parameters for best fitting model to data from Experiment 1.

Condition	Drift		Decision threshold		Non-decision time		Starting point		Ntrials
	M	SEM	M	SEM	M	SEM	M	SEM	M
Low coh	0.060	0.008	0.151	0.013	0.435	0.013	0.076	0.005	614
High coh	0.172	0.008	-	-	0.402	0.016	0.078	0.006	677
Arrows	0.803	0.076	0.214	0.041	0.219	0.010	0.098	0.031	1273
Signal detection	1.056	0.187	0.418	0.107	0.162	0.023	0.265	0.103	199

Data are presented separately for low and high coherence trials (integration conditions), and arrows and signal detection trials (non-integration conditions). The last column indicates the average number of trials in each of the conditions. Parameters that do not vary between conditions are indicated with a dash (-). Our scaling of the threshold parameter adheres to the Ratcliff convention according to which one of the decision thresholds is placed at zero, and the other decision threshold at the value represented by the decision threshold parameter.

**Figure 3:**
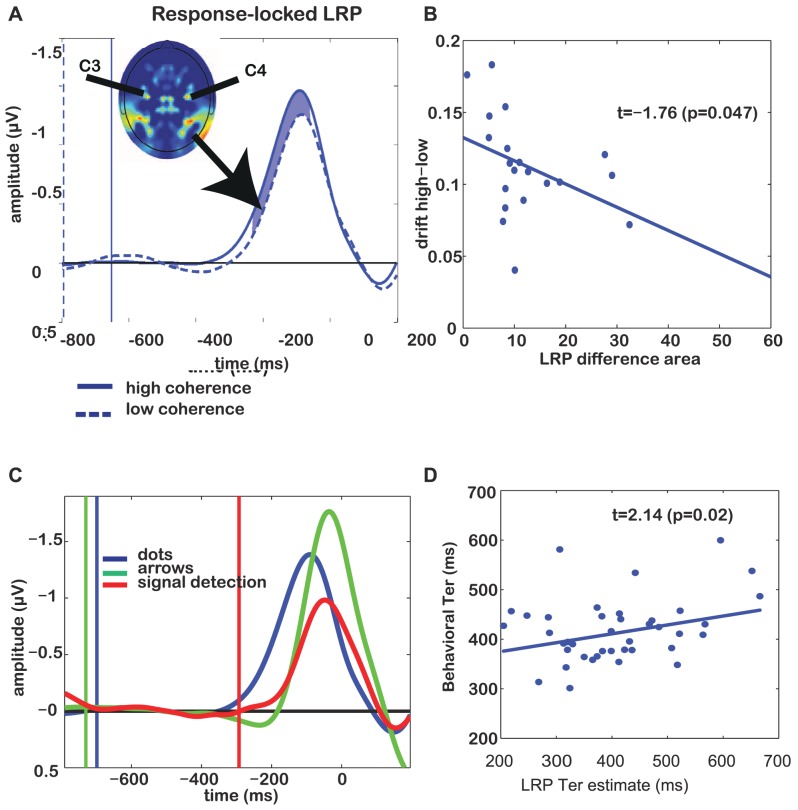
Response-locked LRPs and individual differences for Experiment 1. (A) Grand average response-locked LRP, demonstrating the difference between low and high coherence conditions. Vertical lines indicate stimulus onsets for the respective conditions. Shaded area indicates the time window where low and high-coherence differ significantly (t-test with p<0.05). Inset shows a topographical map (nose up) of lateralized EEG activity, demonstrating that electrodes C3 and C4 are maxima of this measure. (B) Individual differences in DDM estimate of drift rate correlate with area between curves of high- and low-coherence LRPs. Each dot reflects the difference between low- and high-coherence drift and area between LRP curves for a single participant. (C) Grand average response-locked LRP demonstrating the difference between integration and non-integration conditions. Blue trace reflects the evidence-integration condition (average of low- and high-coherence trials). Red reflects a task condition where the participant has to press a pre-specified button, whereas green shows trials on which a participant is instructed by an arrow cue which button to press. Vertical lines indicate dot-motion onsets for the respective conditions. (D) We estimated non-decision time 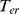 from the LRP by adding the time until departure from baseline to the distance between LRP peak and the actual motor response. The thus-estimated neural 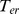 correlates with the behaviorally-estimated 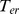. Each dot reflects data from one participant in one condition (low or high coherence).

We next examined whether the LRPs showed evidence of behavior similar to that of threshold units in our model. In particular, the model predicts that, for simple signal detection (which does not require evidence to be accumulated for a discrimination), the activity should ramp up much more quickly than for conditions requiring stimulus discrimination and the integration of evidence (see green time course in Figure 1D). Specifically, the LRP a few hundred milliseconds prior to the ballistic deflection shows clear modulation with changes in the drift rate. In Figure 3C, we compare the response-locked LRPs for trials in which the participant had to integrate motion information to trials in which s/he did not have to do so, because the correct response was specified beforehand (red trace), or indicated with an arrow after dot-motion onset (green trace). Arrow stimuli show a steeper slope than the dot motion condition because their drift rate is higher (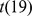 = 2.1, 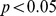; Table 1). This increased drift rate also causes an LRP peak of the non-integration condition (arrows) that is closer to the time of the response than the lower drift rate integration condition (prediction: Figure 1E; experiment: Figure 3C; 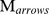 = −28 ms; 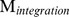 = −92 ms; 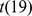 = 11.99, 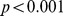).

We then compared the low- and high-coherence response-locked LRPs, the early part of which we predicted would reflect the DDM drift rate. Figure 3A shows that as predicted in Figure 1D, these LRPs differ between low and high coherence in the shaded pre-peak regime. Our model predicts that changing the speed of evidence accumulation should change the shape of the early part of the LRP, which reflects low but increasing levels of input from the accumulator units. Closer to the response, the LRP will look more stereotyped across conditions (see Figure 1D). Consequently, the difference between drift estimates in each motion coherence condition should correlate across subjects with the area between their corresponding LRP curves in their rising phase. It is important to examine the area between curves for the early phase of the LRP, rather than the height of its peak, because our model showed that this early phase is most sensitive to the differences between conditions. We therefore examined whether the area between the curves from 250 to 150 ms before the response, during the LRP's rising phase, reflected the difference between the estimated DDM parameters for low and high coherence (Figure 3B). Indeed, as the area between the low- and high-coherence LRPs increases, the difference between the estimates of the drift rate decreases [robust regression 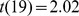, 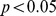].

We next examined whether we could use the response-locked LRPs together with stimulus-locked LRPs to disentangle the stimulus- and response components of the DDM's non-decision time parameter 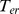. 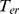 models the combined effects of a perceptual latency and a motor latency. The stimulus-locked LRP shows a period near zero until it departs from baseline (see Figures S4 and S5). We hypothesized that the time until the LRP departs from baseline reflects the stimulus-processing latency, and the time between the peak of the LRP and the response, the motor latency. This follows from our conception of the LRP consisting of two phases: the early, smoothly rising phase as reflecting evidence accumulation, and the later, ballistic phase reflecting the commitment to a decision and actual motor response. We defined the time at which the LRP departed from the baseline as the intersection of the initial horizontal part of the LRP with the rising phase of the peak. Figure 3D shows that 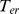 estimated from the LRP in this way correlates with the behaviorally-estimated 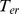 [robust regression, 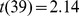, 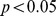]. Together, these results suggest that the early, slowly rising phase of the LRP is consistent with aspects of evidence accumulation that spread from the accumulation layer of our model into its threshold layer.

One may, however, wonder whether the threshold layer of the model is the best fit for the data, or whether alternatively the accumulation layer better models the LRP. To address this question, we computed the root-mean-square deviation (RMSD) between the LRP waveform and the traces predicted by the model (after scaling them to have the same height). For every set of parameters, we then computed the goodness-of-fit between the model-generated averages of its accumulation layer and its threshold layer. The threshold layer showed a better fit (lower RMSD) to the response-locked LRP than the accumulation layer for 59% of the (plausible) parameter space. The plausible parameter space spans those sets of parameters that lead to model responses within the empirically observed response window.

Altogether, Experiment 1 suggests that LRPs exhibit characteristics consistent with our modeled response threshold detectors, which are implemented in bistable, neural switch populations. More precisely, when response-locked, the area between the low- and high-coherence LRPs predicts drift rates of evidence accumulation. In addition, we can predict 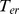 by adding the time to LRP onset to the time between the LRP peak and the actual response. This demonstrates that the LRP may provide a useful index of the average state of evidence accumulation within a trial, despite its typical interpretation as a predominantly motor-related phenomenon (but see [13]). It is also consistent with predictions regarding SAT settings, which is the main phenomenon that the threshold layer of our neural network model aims to describe.

Interestingly, inspection of Figure 3A reveals a notable feature of the data: the LRP for signal detection trials appears to have a smaller peak than the LRP in the conditions in which the response is not known before the start of the trial (i.e., arrows and integration trials). This suggests that the height of the response-locked LRP may also reflect a participant's response bias. When threshold units receive a continuous biasing signal, as would be appropriate in the signal detection case in which only one response is required, they require less evidence input to reach the critical level needed to emit a response. Their pre-stimulus baseline activation levels are already partway toward the threshold for responding. If threshold units are biased asymmetrically in a two-choice context, then the attenuation of the LRP height should be proportional to the DDM's behaviorally-estimated decision bias parameter. Asymmetric biasing is in fact optimal in the two-choice case of Experiment 2, as we describe below [31]. We test this prediction of LRP-amplitude attenuation with increasing response bias in Experiment 2. This is a crucial prediction to test, because up to now, we have only verified the continuous aspect of the threshold units–they reflect signatures of evidence accumulation. The model's ballistic aspect can be tested by examining response bias, which should affect the LRP's magnitude.

### Experiment 2

To test the second prediction from our model—that the height of the LRP peak reflects a participant's decision bias, with a smaller height indicating an increase in decision bias (Figure 1F)—we conducted an experiment in which we manipulated stimulus proportions to induce four distinct levels of decision bias. Simen et al. [40] have previously shown that when the proportions of leftward vs. rightward dot-motion stimuli make it more likely that the dots will move in one of the two possible directions, participants typically adapt to this change, as predicted by Bogacz and colleagues [31], by shifting starting points and reducing thresholds to achieve a nearly optimal response bias.

**Figure 4:**
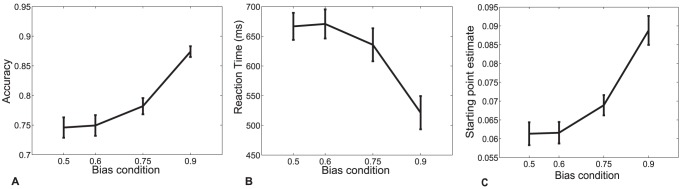
Behavioral data for Experiment 2. Mean accuracy (A) and reaction time (B) as a function of response bias condition. (C). Fitted DDM starting point increases with response bias condition. Response bias condition is operationalized as the proportion of trials in biased direction. Error bars reflect sem.

As expected, participants' accuracies increase and their RTs decrease across blocks as the stimulus proportions in a block increasingly favor one of the two motion directions [see Figure 4; repeated measures ANOVAs indicate that both accuracy and RT vary with the level of response bias: 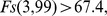 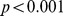], in agreement with previous results [40]. We also note that DDM parameter estimates are sensitive to the different bias-level conditions (Table 2). Given these fits, Figure 4C shows that behavioral estimates of decision bias (as captured by changes in the starting point) increase with the bias condition, as predicted for optimal responding [31].

**Table 2:** DDM parameters for best fitting model to data from Experiment 2.

condition	Drift		Decision threshold		Non-decision time		Starting point		Ntrials
	M	SEM	M	SEM	M	SEM	M	SEM	M
0.50/0.50	0.0952	0.006	0.137	0.006	0.289	0.022	0.0613	0.003	869
0.60/0.40	-	-	-	-	-	-	0.0616	0.003	863
0.75/0.25	-	-	-	-	-	-	0.0689	0.003	880
0.90/0.10	-	-	-	-	-	-	0.0889	0.004	935

Fits were done separately for each of the response bias conditions. Only the starting point is allowed to vary between the different conditions. Parameters that do not vary between conditions are indicated with a dash (-). Last column indicates the number of trials in each condition.

**Figure 5:**
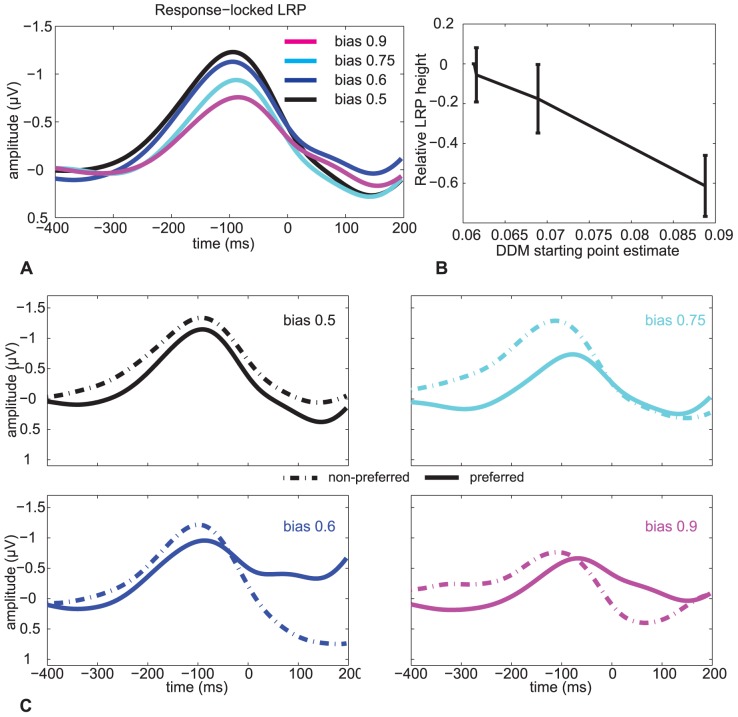
Response-locked LRPs in Experiment 2. (A) Grand average LRP waveform separated by response bias (cf. Figure 1F). (B) Relationship between normalized LRP peak height and fitted DDM response bias. There is a significant correlation between the DDM starting-point parameter and LRP height. Error bars reflect sem. (C) Grand average response-locked LRPs in Experiment 2 separately for the preferred (solid) and non-preferred (dashed) direction. As predicted by our model, the LRP is larger for the non-preferred compared to the preferred direction [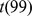 = 11.4, 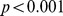].

Figure 5A shows a grand average response-locked LRP split by bias condition. The height of the LRP decreased with decision bias [one-way repeated measures ANOVA with response bias probability as continuous variable 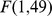 = 25.4, 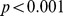]. We next asked whether decreases in peak height within participants correlated with individual differences in the behaviorally-estimated response bias. When we subtracted peak height from the height of the LRP in the unbiased condition, there was a significant relation between this normalized LRP height and behaviorally-estimated DDM starting points [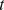-test on individual participant slopes of LRP height on starting-point estimate 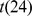 = 5.74, 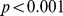; see Figure 5B].

If the height of the LRP reflects the distance from threshold, which decreases with response bias, then this also predicts that the LRPs for the trials in which the stimuli move in the direction opposite to the bias direction (“non-preferred”) should have a higher amplitude. Figure 5C shows that indeed this is the case [paired 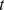-test on areas between the preferred and non-preferred curves between −150 and −50 ms: 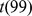 = 11.4, 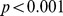]. In short, the dynamics of the LRP are also consistent with our model's predictions for manipulations of response bias.

## Discussion

There is a substantial literature on the neural basis of the integrators that correspond to the accumulation layer of the model (Figure 1A). In contrast, thresholds play a critical role in nearly all decision making models–allowing levels of evidence (or urgency, or preference) to rise and fall during deliberation without any concomitant bodily movement prior to decision commitment. Yet, the mechanisms underlying threshold operation have received far less attention than the mechanisms underlying evidence accumulation [18]. We have therefore focused on finding non-invasive, high temporal resolution signatures of a neural threshold mechanism in the context of a three-stage neural network model of the decision process that specifies a neurally-plausible mechanism for crossing the decision threshold.

We have shown that LRPs may reflect the operation of such mechanisms. We found that LRP dynamics are consistent with the dynamics predicted by our model's threshold layer. In particular, the early non-ballistic LRP component's behavior reflected signal-to-noise ratio. The LRP's shape was more consistent with activation levels in the threshold layer of our neural network model than with those in the accumulation layer. We also found that neural correlates of 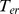 (non-decision time) were correlated with behavioral estimates of 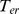. The magnitude of the ballistic part of the LRP (peak height) was consistent with predictions concerning response bias.

### Peak shifting

Beyond these basic predictions of the model, we have given a model-based account of LRP peak-shifting between integration (motion-detection) and non-integration (arrows/signal detection) conditions of our task. Previous studies [41]–[44] have not produced a clear picture of which task conditions cause a shift in LRP peak. We have demonstrated that the LRP peak moves closer to the response when there is a very high drift rate, and the threshold unit rises to a maximum before resetting (Figure 1E). However, in addition to our account, elements of these other accounts may also contribute to peak shifting. For example, when the evidence is less noisy (in the non-integration trials), it may produce more rapid and ballistic post-decision motor processing because the participant may allow less continuous checking of the to-be-emitted response [45].

### Does the LRP reflect evidence accumulation or threshold crossing?

Historically, the LRP has played an important role in debates about the nature and timing of mental processing (e.g., [35], [46], [47]). In the context of decision making, for example, Spencer & Coles (1999) [33] showed that the LRP recordings of Gratton et al (1988) [46] qualitatively matched the activity of evidence accumulator units in a neural network model of the flanker task [48], at least during the non-ballistic phase of the LRP. Rinkenauer et al (2004) [35] focused on the LRP as a signature of SAT adaptation, but argued that their data were inconsistent with models of evidence accumulation. We propose that the early part of the LRP *does* reflect evidence accumulation (and therefore correlates with individual differences in DDM parameters), whereas variations in the later, ballistic part, on which Rinkenauer and colleagues focus, reflect primarily the adaptation of SAT. According to our model, the slowly rising phase of the LRP results from the sub-threshold input to the threshold units by the accumulator units. Once threshold unit activity crosses a critical level, it rises very quickly to a maximum, which accounts for the ballistic phase of the LRP. Our proposal is similar to that of Kelly & O'Connell [49], who recently showed that the LRP occurred at a later point in time than a putative correlate of evidence accumulation (in their account, the centroparietal positive potential).

### Fixed vs. modifiable thresholds

Evidence for threshold mechanisms in neural activity is abundant, and adapting thresholds to change SATs is a classic element of psychological decision making models [1]. However, the degree to which thresholds are modifiable is disputed on the basis of physiological evidence. Purcell et al. (2010) [28], for example, examine ramping response-locked FEF movement/buildup neuron activity in monkeys responding to visual stimuli with saccadic eye movements. Although movement neurons in the FEF are arguably at a neural level analogous to our threshold layer, they model this activity as a process of gated evidence accumulation our accumulation layer), in which evidence accumulation begins only after a threshold level of input from a sensory detector (visual neurons in FEF) is received. RTs are determined by the time at which evidence exceeds a second, fixed, response threshold, and the slope of this evidence is a key determinant of RT, although in their work the ramp-up period typically occupies a small proportion of the total RT.

This work by Purcell, Schall and colleagues adds to a body of physiological data that appears to provide evidence against the notion of easily modifiable response thresholds. FEF activity in experiments such as [28], [50] typically ramps to the same level regardless of RT. In our Experiment 2, we have interpreted our data in precisely the opposite terms, with biasing activity hypothesized to modify the level to which evidence should accumulate before triggering a response (by supplementing the evidence inputs to the threshold units). This is also consistent with recent work by Heitz & Schall (2012) [51], who demonstrated there were neurons in motor cortex (recorded during a visual search task) that had a different final firing rate depending on the level of their SAT. It should, however, be noted that they found several other signatures of SAT adaptations as well.

Some evidence suggests that instead of thresholds themselves, it is the initial firing rate at which putative accumulators begin to accumulate evidence that effects a change in response biases [52]. This type of dynamics might also account for our LRP data, but it would require biasing the accumulator layer of our model rather than the threshold layer (see e.g., [53], [54]). To still approximate optimal evidence accumulation in such a model, the biasing signal would need to be disabled at the start of each accumulation [31]. Our model of the threshold layer provides another, possibly simpler way to resolve the conflict between behavioral evidence for threshold adaptability and optimal evidence accumulation versus physiological evidence for fixed response thresholds. If putative FEF evidence accumulators in Purcell et al. [28] are instead reinterpreted as threshold-crossing detectors, then the signatures of evidence accumulation they display can be understood as the echoes of evidence accumulation taking place in other brain areas that project to FEF — areas in which accumulators may in fact ramp to different levels of activity before triggering transitions from an FEF switch's down state to its up state.

The reason that the neural data are often taken to support fixed thresholds is that adjustable thresholds are difficult to observe in neural data. A simple modification of our model illustrates why this is the case. Adjustable decision thresholds might be obscured by neurally plausible excitatory feedback connections from the threshold layer to the accumulator layer. These would force the accumulators up to the same level for all decisions, following the stereotyped behavior of the threshold units. Hence, indications that a given brain area plays a role in evidence accumulation should focus on the earliest levels of neural processing at which ramping can be observed (see [3] for more details).

### Non-decision time

In our study, we observed a relation between LRP peaks and behavioral non-decision time (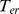; Figure 3D). The slope of this relationship was smaller than one, such that the neurally-estimated 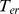 underestimates the behaviorally-estimated 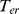. It is therefore likely that in addition to the perceptual and motor processes that we estimate from the LRP, there is at least one other process that contributes to 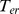. An alternative possibility is that the small slope is caused by the fact that the 1DF method that we use to determine the LRP-based estimate of 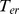 is biased towards finding shorter 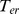 estimates, because the earliest above-baseline fluctuation will determine 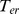. As a result, the slope between behaviorally- and LRP-estimated 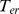 will tend to be smaller than one, as we observed.

### Effects of bias

The outcome of our bias manipulation is consistent with the results of previous LRP studies that did not explicitly investigate connections to the DDM. Jentsch and Sommer (2002) [55] saw shallower LRPs for repeat- compared to alternation trials, and it is plausible that repeat trials create a decision bias (e.g., [56], [57]). Töllner and colleagues [58] similarly showed that, relative to the preceding choice, a different response in a visual search task has a larger amplitude than the same response. Scheibe and colleagues [59] showed a larger LRP amplitude for invalidly cued compared to validly cued trials. A cue will make a response in the cued direction more likely, which, in a DDM framework, would be modeled by moving the starting point closer to the relevant decision threshold on those trials [59]. did not observe an effect of explicitly manipulated prior probability of a response on LRP amplitude, although they might have observed one if they had included the most extreme prior probability conditions. In short, our work shows that these previous findings of changes in LRP amplitude can be reinterpreted as changes in DDM starting points.

## Conclusion

We have shown evidence consistent with the hypothesis that the LRP derives from the activity of threshold units in a neural network implementation of the DDM. Our model provides a neural mechanism for turning a continuous signal into a discrete output. Because the threshold units lie in-between evidence accumulation units and motor output units, LRPs exhibit both an early signature of gradual evidence accumulation as well as a later, ballistic, response-related (“motor”) component. An example of evidence for the gradual-accumulation interpretation of the LRP is that the area between curves from the low- and high-coherence conditions correlates with individual differences in behaviorally-estimated DDM parameters. Similarly, the height of the LRP agrees with predictions of changes in response bias. We therefore suggest that the LRP signals the process of crossing a decision threshold, but also, because it provides an echo of the process of evidence accumulation, that it can be used to study how experimental manipulations affect evidence accumulation in the brain.

## Materials and Methods

### Ethics statement

The experiments were approved by the Institutional Review Board of Princeton University. The procedure included written informed consent, which was provided by all participants.

### Experiment 1

#### Model

Matlab code used to simulate the neural network model that made the qualitative predictions is available in File S2.

#### Task

In this experiment (for which some of the data have also been reported in [12]), we tested the model's prediction that manipulating stimulus coherence would change the area between the LRP curves for low- and high coherence conditions. Participants determined the motion direction of random dot kinematograms. These random dot kinematograms were similar to those used in a series of psychophysical and decision making experiments involving humans and monkeys (e.g., [5], [39], [40], [60], [61]). Stimuli consisted of an aperture of approximately 7.6 cm diameter viewed from approximately 100 cm (approximately 4 degrees visual angle) in which white dots (2×2 pixels) moved on a black background. A subset of dots moved coherently either to the left or to the right on each trial, whereas the remainder of dots were distractors that jumped randomly from frame to frame. Motion coherence was defined as the percentage of coherently moving dots. Dot density was 17 dots/square degree, selected so that individual dots could not easily be tracked. Tracking was further discouraged by using three interleaved sets of dots of equal size, each of which was plotted in one of three successive video frames. Therefore each set of dots returned after three frames, with a random displacement. The speed of the dots was approximately 7 degrees/second.

Following the procedure used in [40], stimuli remained visible until participants made a response (i.e., pressing the ‘Z’ key with their left index finger to indicate leftward motion or the ‘M’ key with their right index finger to indicate rightward motion), at which point the stimulus disappeared and a variable response-to-stimulus interval (RSI) ensued. Correct responses were rewarded with $0.01; errors were unrewarded. Sessions lasted a fixed amount of time (50 minutes), so faster performance led to more trials completed. Participants made on average $12.27 per session. Reward feedback was displayed visually and signaled with a tone after each trial. Participants were instructed to maximize their earnings.

The arrows control condition was constructed as follows. Each arrows trial started with random dot motion (0% coherence), followed by the appearance of a clearly visible yellow arrow in the center of the screen pointing in the direction to which a participant should respond. The onset time of the arrow was calibrated such that the duration of 0% coherence dots viewing time matched the dot-viewing times in the main task. More concretely, the arrow onset time distribution was created by subtracting the average button-press latency (obtained from the signal detection control task) from a randomly selected RT in the main task from the previous session (this was done separately for each coherence). We restricted our EEG analyses to the correct trials, because there are many different reasons for making errors, which could introduce noise in the model predictions and analyses.

The experiment presentation code was written in PsychToolbox [62]. Dot stimuli were presented with PsychToolbox extensions written by J. I. Gold (http://code.google.com/p/dotsx/).

#### Participants

Twenty-three members of the Princeton community (twelve female, mean age 25) participated in Experiment 1. Participants engaged in three hour-long behavioral-only training sessions in which they became familiar with the task. At the beginning of these training sessions, performance on a psychometric block (trials with fixed viewing times of 1000 ms and five different coherences) was used to determine the coherences at which they performed at approximately 70 and 90% correct. These coherence levels were used for the remainder of the session, and the coherences from the last psychometric block were used for the two EEG sessions. Data of three participants, whose LRPs did not show any modulation by movement, were removed from the analysis.

#### Recording Methods

We recorded EEG data from 128 channels using Neuroscan EEG caps (Neuroscan, Charlotte, NC) with a Sensorium EPA-6 amplifier (Sensorium Inc., Charlotte, VT). Data were digitized at 1000 Hz and band-pass filtered from 0.02–300 Hz; all impedances <30 kΩ. Acquisition was controlled by Cogniscan software (EJC Systems Inc., Newfoundland, NJ). All data were referenced to the left mastoid and off-line rereferenced to an average reference after automatic bad-channel removal [63], [64].

#### Data Analysis

The LRP is thought to reflect the lateralized aspect of the activity of primary motor cortex [46], [47], [65], [66] and is computed by subtracting the EEG in electrode C3 from electrode C4 for left-handed responses, and C4 from C3 for right-handed responses, and then averaging these differences [67]. Figure 3A, which shows a topography of lateralized EEG activity, demonstrates that C3 and C4 are indeed the maxima of lateralized response activity.

We computed LRPs with 4-Hz low-pass filtered EEG data [35]. Note that the results with higher low-pass filter cut-offs are qualitatively similar; importantly the correlations of aspects of the LRPs with drift diffusion model parameters (discussed in more detail below) are still significant (p<0.05) with a low-pass filter cut-off of 40 Hz. Trials with eyeblinks (detected with a running average of the eye channel exceeding 100 µV) were removed. We also removed trials with signal amplitude larger than 70 µV, or variance larger than 80 or smaller than 0.1 µV, or kurtosis larger than 5 [68] from the analysis. Stimulus-locked LRPs were baseline-corrected to the average over the 200 ms period immediately preceding stimulus-onset. Response-locked LRPs were baseline-corrected to the period of 400–600 ms pre-response [35]. All LRP plots in this article are grand averages, i.e., averages across all participants.

We determined the LRP height for stimulus-locked LRPs by computing the distance between the height of the LRP at its onset and the peak (the first maximum before the RT). LRP onset was defined by 1DF (1-degree-of-freedom) regression [69], [70], and verified by visual inspection. 1DF regression finds the intersection between a line fitted to the stimulus onset (the initial segment) and a line fitted to the LRP rise to peak. We constrained the slope of the initial segment to be zero.

#### Model fits

We fit the DDM to the behavioral data for each individual participant with the diffusion model analysis toolbox (DMAT; [37], [38]). As demonstrated by Bogacz [31], the DDM closely approximates the accumulator layer of the neural network model of Simen and Cohen [2]. DMAT captures individual differences in drift rate, speed-accuracy trade-off and bias sensitivity. These translate into testable predictions regarding threshold unit dynamics that we examine here. The version of the model we fit is the pure DDM [71], in which there is no variability in drift rate, starting point, or non-decision time.

### Experiment 2

The methods for this experiment were identical to those described for Experiment 1, with the exception of the following.

#### Task

In this experiment, we used only a single coherence level (corresponding to 80% correct performance), while we varied response bias. Response bias was manipulated by changing the probability that the dots would move in one of the two directions from 0.5 (no bias) to 0.6, 0.75 and 0.9. We also manipulated response-stimulus interval (RSI; see [40] for a review of the effects of RSI and prior probability on behavioral performance in two-alternative forced-choice tasks with response-terminated stimuli). For the purposes of this analysis, we collapse across the two RSI levels, which did not show any consistent differences in this behavior. Also in this experiment we focused exclusively on the correct trials, which was even more important than in Experiment 1, because the error rates differed between conditions.

#### Participants

Twenty-five members of the Princeton community participated in Experiment 2 (fifteen female, mean age 20.1). In this experiment, we adapted each participant's motion coherence during the practice sessions such that they performed at approximately 80% correct. Participants were trained for four hour-long behavioral sessions. Participants made on average $16.02 per session.

#### Data Analysis

We determined the height of response-locked LRPs by computing the distance between the last peak before the response and the preceding trough. We verified the output of this automated procedure by visual inspection. When we computed the areas between curves we chose a time window identical to the window used in Experiment 1.

## Supporting Information

Figure S1 **Mechanism for threshold crossing detection predicted by the model's threshold layer.** (A) Small arrows and shading depict the rate of change of a threshold unit's activation as a function of its current input and its own output. Dark shading implies negative, light shading implies positive velocity. Solid S-curve depicts stable equilibrium points of the noise-free system; dashed segment of the S-curve depicts unstable equilibria; dark circle depicts a typical initial condition. Arrows depict the velocity of the system state, and are vertical because input is constant. (B) Small vector field arrows show a rightward component when input is positive and increasing. Large arrows show the state trajectory: gradual increase in subthreshold region, followed by rapid increase in the unstable region, resulting in the rising phase shown in Figure 1B. (C) When threshold layer output enters superthreshold region, response layer units abruptly activate, supplying inhibition that forces the threshold unit back down to its initial value (see Figure 1C).
(EPS)

Figure S2 **Observed versus expected quantiles of the RT distribution, illustrating model fit quality for Experiment 1.** A perfect fit would show all points on the unit slope line. Different symbols reflect the different drift conditions.
(EPS)

Figure S3 **Observed versus expected quantiles of the RT distribution, demonstrating model fit quality of Experiment 2.** Shown are from left to right, top-to-bottom the 10th, 30th, 50th, 70th and 90th quantile. A perfect fit would show all points on the unit slope line. Different symbols reflect the different bias conditions.
(EPS)

Figure S4 **Grand average** ***stimulus-locked LRPs*** **for Experiment 1, emphasizing the difference between integration and non-integration conditions occurring close to stimulus appearance.** Vertical lines indicate median RT for each condition. Note that the LRP for the arrow trials (green) rises only relatively late because the arrow that indicates the response arrives after a period of dot motion. The arrow arrival time is calibrated to create dot-viewing times equivalent to the dot-motion trials.
(EPS)

Figure S5 **Stimulus-locked LRPs and individual differences for Experiment 1.** (A) Grand average LRP waveforms, separated by coherence. Vertical lines indicates median RT for the respective conditions. Shaded area indicates the time window for computing the area between curves. The LRP rises more quickly for high- than for low-coherence conditions. (B) Difference between low- and high-coherence stimulus-locked LRP in the window from 400–500 ms post-stimulus correlates with individual differences in drift rate. Each dot reflects the difference between low- and high-coherence LRP and drift rate for an individual.
(EPS)

File S1 **Supplementary information.** More detailed description of the model, stimulus-locked LRP data, and model fit quality assessments.
(PDF)

File S2 **Model code.** Code to fit the neural network model of decision making of Simen and Cohen (2009).
(ZIP)
