# Correction: Lateralized Readiness Potentials Reveal Properties of a Neural Mechanism for Implementing a Decision Threshold

**DOI:** 10.1371/journal.pone.0132197

**Published:** 2015-06-29

**Authors:** Marieke K. van Vugt, Patrick Simen, Leigh Nystrom, Philip Holmes, Jonathan D. Cohen

The authors made a computational error in their analyses, such that they reported correlations based on the absolute value of the area between LRP curves rather than the signed value. The model they evaluated, however, makes predictions about the signed value of this area, not its absolute value. When the curves are compared using the correct, signed values of this area measure, the correlation is no longer significant (response-locked r(19) = 0.18, t(19) = 0.93, p = 0.183; for the supplementary data, stimulus-locked r(19) = -0.23, t(19) = 1.44, p = 0.083).

Implications: The article claims that LRPs reflect echoes of evidence accumulation as well as decision threshold heights and response biases, and are not merely signals of motor program execution. Even in the absence of Fig 3B, the main conclusions of the article still hold. Specifically, although individual differences in drift rate do not correlate with changes in the LRP within subjects, as originally thought, there is still evidence that LRPs reflect echoes of evidence accumulation. For example, the erroneous analysis does not affect the original finding of a shift in the peak location of the LRP with very high levels of drift (the "arrows task" produces both high drift and a shift of LRP later in time). Neither does this error affect the original findings of correlations between the onset time/peak time of the LRP and the behaviorally estimated non-decision time for each participant. In line with the primary claim that LRPs reflect the operation of a decision threshold mechanism, the original findings of correlations between the magnitude of response bias and the height of the grand average LRP are unchanged.

Please see below for a summary of the errors and their corrections:

Fig 3B should be removed from the figure. The authors have provided a corrected version of Fig 3 and its caption here.

**Fig 3:**
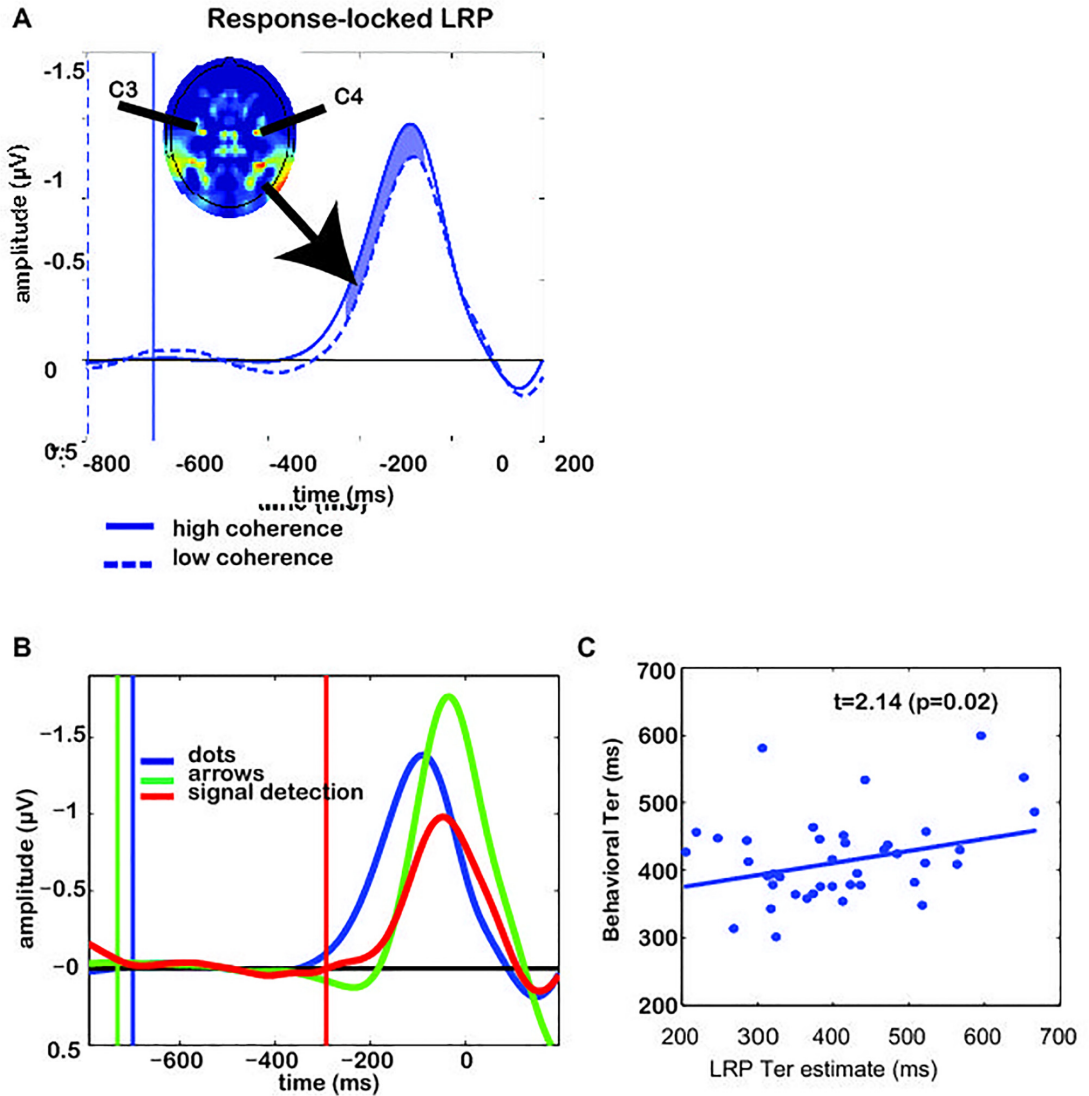
Response-locked LRPs and individual differences for Experiment 1. (A) Grand average response-locked LRP, demonstrating the difference between low and high coherence conditions. Vertical lines indicate stimulus onsets for the respective conditions. Shaded area indicates the time window where low and high-coherence differ significantly (t-test with p<0.05). Inset shows a topographical map (nose up) of lateralized EEG activity, demonstrating that electrodes C3 and C4 are maxima of this measure. (B) Grand average response-locked LRP demonstrating the difference between integration and non-integration conditions. Blue trace reflects the evidence-integration condition (average of low- and high-coherence trials). Red reflects a task condition where the participant has to press a pre-specified button, whereas green shows trials on which a participant is instructed by an arrow cue which button to press. Vertical lines indicate dot-motion onsets for the respective conditions. (C) We estimated non-decision time *T*_er_ from the LRP by adding the time until departure from baseline to the distance between LRP peak and the actual motor response. The thus-estimated neural *T*_er_ correlates with the behaviorally-estimated *T*_er_. Each dot reflects data from one participant in one condition (low or high coherence).

There is an error in the sixth sentence of the Abstract. The correct sentence is: We show that, as our model predicts, LRP amplitudes and growth rates recorded while participants performed a motion discrimination task correlate with individual differences in behaviorally-estimated prior beliefs, decision thresholds.

In the sixth paragraph of the “Experiment 1” subsection of the Results, the following sentence should be removed: Indeed, as the area between the low- and high-coherence LRPs increases, the difference between the estimates of the drift rate decreases robust regression t(19) = 2.02, p<0.05].

There are errors in the second sentence of the ninth paragraph of the “Experiment 1” subsection of the Results. The correct sentence is: When response-locked, the LRPs significantly differ in amplitude between low- and high levels of accumulated evidence.

There are errors in the third sentence of the second paragraph of the Discussion. The correct sentence is: In particular, the early non-ballistic LRP component's amplitude was modulated by the signal-to-noise ratio.

There are errors in the fourth sentence of the Conclusion. The correct sentence is: An example of evidence for the gradual-accumulation interpretation of the LRP is that the onset and offset times of the LRP can together be used to predict an individual's non-decision time.

S5 Fig contains errors. Please view the correct S5 Fig below.

## Supporting Information

S5 Fig Stimulus-locked LRPs and individual differences for Experiment 1. Grand average LRP waveforms, separated by coherence. Vertical lines indicates median RT for the respective conditions. Shaded area indicates the time window for computing the area between curves. The LRP rises more quickly for high- than for low-coherence conditions.
(TIF)
