# Supplementary material for: Lateralized Readiness Potentials Reveal Properties of a Neural Mechanism for Implementing a Decision Threshold
Source: PLoS One. 2014 Mar 13;9(3):e90943. doi: 10.1371/journal.pone.0090943 (PMC3953213; doi:10.1371/journal.pone.0090943)
Supplement: File S1 — Supplementary information. More detailed description of the model, stimulus-locked LRP data, and model fit quality assessments. (PDF) [file pone.0090943.s006.pdf]

Supplemental Materials - Detailed model specification and model fits

M.K. van Vugt, P. Simen, L. Nystrom, P. Holmes, J. D. Cohen

Detailed model presentation

Model units

The model consists of units that represent the firing rate V of entire neural populations.

We assume the inputs to a population have Gaussian white noise added to them (Wiener process W), which models the input variability introduced by stochastic spike-generation in the input populations, and is weighted by a factor c. $V(t)$ can thus be interpreted as the dynamic rate parameter of a non-homogeneous Poisson process; it is defined as follows, with the subscript i denoting 'units' representing distinct populations, and w_{ij} denoting entries of the connection matrix linking units into a network:

$$\tau \cdot dV_i(t) = \left[-V_i(t) + f\left(\sum_j w_{ij} V_j \right) \right] \cdot dt \; + \; c \cdot dW_i, \quad (1)$$

$$f(I) = \frac{1}{1 + \exp(-\lambda(I - \beta))}. \quad (2)$$

These equations imply that for a constant input I, the resulting population activity at time t, $V(t)$, exponentially approaches $f(I)$ (Figure S1). The activation function f is a sigmoidal function of its inputs: a nearly linear function over part of its input range that saturates at a maximum firing rate for very high input levels, and at a minimum of zero for very low input levels.

The model is able to reproduce the slow rise of an evidence accumulator as well as the bistable, ON-OFF switching behavior that defines a threshold unit. Bistability, which allows the two discrete states of decision-preparation and decision-commitment to be represented unambiguously (see [1]), arises as follows. In Eq. 2, β reflects the input level that produces 0.5 output activation in a unit. When recurrent self-excitation w_{ii} equals 0, the activation function f defines the equilibrium level of output activation for a given input. In contrast, with $w_{ii} > 0$, the equilibrium output level is higher than $f(I_0)$ for an input of size I_0, since the net input is now $I_0 + w_{ii}V_i$. The activation function has effectively steepened.

When w_{ii} exceeds the critical value $\lambda/4$, the resulting activation function mapping inputs to outputs folds back on itself. This function is depicted as a black, S-shaped curve in the three panels of Figure S1. Folding of the activation function implies that a single unit's activation is bistable in the input range over which the S-curve is dashed rather than solid: arrows and shading denote that for any input in this range, the output approaches the upper solid curve if it starts out above the dashed portion, and the lower solid curve if it starts out below the dashed portion. Bistability in turn implies that the unit can act as an ON-OFF switch, and therefore as a threshold-crossing detector, like ON-OFF switches in commercial electric circuits [2]. If such a unit begins at zero input and near zero output (black circle in FigureS1A), then receives input that increases over time (Figure S1B), its input-output trajectory (large black arrows) will eventually cross into a region with only a single, stable level of activation near the maximum possible firing rate. At this point, feedback inhibition can drive the system back to the origin (Figure S1C).

Model layers

The model consists of three layers that are described in more detail in the main text. The hysteresis mechanism described above crucial for the threshold and response layers. For the threshold units, hysteresis prevents them from "falling back" after they have just crossed the threshold. For the response units, hysteresis allows them to completely reset the preceding decision layers. It imposes a sufficiently long delay between threshold-unit activation and subsequent reset-switch activation to ensure that decision signals from threshold units are not silenced prematurely. It furthermore causes a delay from when the threshold unit shuts off to when the reset signal itself shuts off; this delay can be made long enough to ensure complete resetting of all units to zero activation.

In brief, the accumulation layer primarily serves to accumulate information; the response layer is needed to convert the binary decision into a motor response and to reset the threshold layer, and in-between that is the threshold layer that converts a continuous evidence accumulation process into a binary decision in a neurally-plausible manner. Matlab code used to simulate the model is available online with this article.

Quality of model fits

We estimated evidence accumulation parameters for each individual by fitting the drift diffusion model to their behavioral data. To assess the quality of these DDM model fits, we plotted the quantiles of the predicted RT distribution against the empirically observed RT distribution. A perfect fit would have all datapoints (each point reflecting the RT quantile for each participant in a particular condition) on the black unit-slope line. Figure S2 shows that the fits are close, though slightly worse for the 90th percentile than for the other percentiles, as is often the case with the DDM (see, e.g., [3]).

Similarly, Figure S3 shows that as in Experiment 1, fits are close, and the biggest deviations occur in the least stable, 90th percentile plot.

Model predictions for stimulus-locked LRPs

While we focus in the main text on verifying model predictions with response-locked LRPs, one can also consider stimulus-locked LRPs. Stimulus-locked LRPs are best suited to reveal perceptual processes that are strictly time-locked to stimulus onset, whereas response-locked LRPs reveal motor- and response-related processes. Nevertheless, we predict that in both types of LRPs, signatures of evidence accumulation should be visible. We will use the stimulus- and response-locked LRPs together to predict non-decision time.

Figure S4 shows that, as expected, in the signal-detection trials in which the participant must only detect the onset of dots stimuli but is not required to accumulate evidence about motion direction, the stimulus-locked grand average LRP increases more steeply than in the motion discrimination conditions.

The model also predicts that when the evidence that is being accumulated is noisier, the drift rates are lower (see Table 2 in the main text) and hence the LRP for low-coherence trials (low drift) should remain at baseline for longer than the LRP for high-coherence trials. Figure S5A shows that there is a difference in the rate of LRP rise between low- and high- coherence trials (compare to Figure 2 in the main text). Furthermore, as in Figure 2, the peak of the low-coherence condition is lower than that of the high-coherence condition. This is caused primarily by greater temporal smearing of the LRP peaks in the low-coherence case due to greater RT variability.

We then correlated the DDM drift parameter with the area between the low and high-coherence curves of the stimulus-locked LRP between 400 and 500 ms post-stimulus. This time interval was chosen based on visual inspection of the waveforms. The area between the curves (Figure S5A) correlates with each individual's behaviorally-estimated drift value [robust regression, $t(19) = 1.81$, $p < 0.05$; Figure S5B].

References

1. Simen P (2012) Evidence accumulator or decision threshold – which mechanism are we observing? Frontiers in Psychology 3: 183. doi: 10.3389/fpsyg.2012.00183.

2. Dorf RC, Svoboda JA (2006) Introduction to Electric Circuits. Wiley, 7th edition.

3. Ratcliff R, Frank MJ (2012) Reinforcement-based decision making in corticostriatal circuits: Mutual constraints by neurocomputational and diffusion models. Neural Computation 24: 1-44.
