# Supplementary figures and images for: Correction: Lateralized Readiness Potentials Reveal Properties of a Neural Mechanism for Implementing a Decision Threshold
Source: PLoS One. 2015 Jun 29;10(6):e0132197. doi: 10.1371/journal.pone.0132197 (PMC4488310; doi:10.1371/journal.pone.0132197)

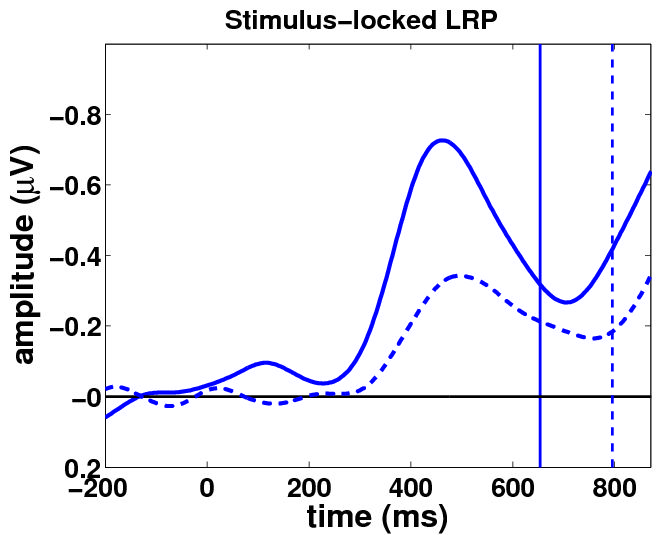

Supplement: S5 Fig — Grand average LRP waveforms, separated by coherence. Vertical lines indicates median RT for the respective conditions. Shaded area indicates the time window for computing the area between curves. The LRP rises more quickly for high- than for low-coherence conditions. (TIF) [file pone.0132197.s001.tif]
